# Turn‐Taking and Vocal Coordination in Mother–Child Mixed‐Hearing Dyads and the Effect of Home Music Engagement: A Longitudinal Study on Italian Children With Cochlear Implants

**DOI:** 10.1111/1460-6984.70243

**Published:** 2026-04-17

**Authors:** Valentina Persici, Letizia Guerzoni, Domenico Cuda, Marinella Majorano

**Affiliations:** ^1^ Department of Human Sciences University of Verona Verona Italy; ^2^ Department of Humanities University of Urbino Carlo Bo Urbino Italy; ^3^ Otorhinolaryngology Unit ‘Guglielmo da Saliceto’ Hospital Piacenza Italy; ^4^ Medicine and Surgery Department University of Parma Parma Italy

## Abstract

**Objectives:**

Studies have shown that the amount of turn‐taking and vocal coordination in mother–child dyads, as well as amount of home music engagement, have important effects on children's language and social development. However, relatively few studies have examined the development of vocal behaviours in mixed‐hearing and hearing dyads, and no study to our knowledge has examined the potential effect of amount of home music engagement on dyadic turn‐taking and timing in mixed‐hearing dyads.

**Design:**

This study examined these aspects in 34 mother–child dyads speaking Italian (17 hearing dyads and 17 dyads with children with cochlear implants [CIs]) across two time points, corresponding to before implantation (T1; mean age = 17 months) and to one year after CI activation (T2) for children with CIs. Mother–child interaction was videorecorded and coded offline. For both mothers and children, we extracted the following vocal behaviours: relative frequency of temporally contingent responses and of simultaneous speech, and timing of within‐ and between‐speaker pauses. Amount of home music engagement was assessed using a parent report.

**Results:**

Results showed that the amount of turn‐taking and degree of coordination between partners changed over time and was influenced by children's hearing impairment. We found reciprocal influences between mothers and children in their pause timing, with mothers seemingly guiding children towards the appropriate interactional timing, while remaining sensitive to their child's vocal behaviour. Finally, we found that greater music engagement at home was associated with more fluid and stable interactional exchanges in mixed‐hearing dyads.

**Conclusions:**

Our findings highlight how turn‐taking and its timing are affected by developmental trends, language skill, and hearing abilities. Importantly, they also show that children with CIs are sensitive and reactive to their mothers’ vocal behaviour, and that the timing characteristics of turn‐taking in mixed‐hearing dyads are predicted by their amount of music engagement at home. These results suggest that everyday musical experiences may support the development of communicative timing in the early interactions of mixed‐hearing dyads, and highlight the potential of music‐based interventions in supporting language and communicative development for children with CIs.

**WHAT THIS PAPER ADDS:**

*What is already known on this subject*

- Turn‐taking and vocal coordination in mother–child interactions are critical for children's language and social development. Home musical engagement has also been linked to improved language outcomes and coordination skills. Children with cochlear implants (CIs) show sensitivity to the timing of early mother–child interactions. However, few studies have compared the development of vocal behaviours in mixed‐hearing dyads (hearing parent and child with CI) to those in hearing dyads, and the findings to date have been mixed. To our knowledge, no study has explored whether turn‐taking and timing in mixed‐hearing dyads are positively influenced by greater amounts of home music engagement.

*What this study adds to existing knowledge*

- This longitudinal study is the first to investigate turn‐taking and timing in Italian‐speaking mixed‐hearing dyads over time (from before implantation to one year after CI activation) and in relation to home music engagement. This study confirms previous research showing that turn‐taking becomes more frequent and temporally stable with age and increasing language proficiency, and that mothers and children influence each other's timing. In addition, it shows that some temporal characteristics of the interaction are negatively affected by children's hearing impairment. Finally, it shows that greater home music engagement is associated with more stable and fluid interactions in mixed‐hearing dyads.

*What are the actual clinical implications of this study?*

- The findings suggest that mothers’ vocal behaviour characteristics play a role in shaping children's vocal coordination skill development and that everyday musical activities may enhance communicative timing in children with CIs and their mothers, supporting smoother early interactions. This opens new avenues for music‐based interventions targeting language and social development in populations at risk for communicative difficulties, including those with hearing impairments.

## 1 Introduction

### 1.1 Mother–Child Vocal Coordination in Interaction

In conversations, adults produce input that is multidimensional and temporally coordinated across several levels. Conversational partners temporally align their gestures, facial expressions, and vocalizations and adults usually produce minimal gaps and overlaps between conversational turns (Levinson and Torreira 2015; Sacks et al. 1974). Such coordination implies that adults are able to comprehend and integrate auditory and visual cues from the other conversational partner, predict when they are finished, and plan and produce their response in a timely manner (Levinson and Torreira 2015). Such precise prediction and coordination mechanisms are possible because conversations are rhythmically organized, just like many other motor behaviours, and their tempo is shared between conversational partners (Jaffe et al. 2001).

Children with normal hearing (NH) begin to actively participate in proto‐conversations with their caregivers very early on, well before they attain actual linguistic competence (Jasnow and Feldstein 1986). Infants are able to alternate their vocalizations with those of their caregivers and their production of pauses between their vocalizations (i.e., within‐speaker pauses) suggest that they are sensitive to the temporal structure of the interaction (Gratier et al. 2015; Hilbrink et al. 2015; Kondaurova et al. 2019). However, while adults are typically skilled at coordinating their vocal behaviours with those of their conversational partner, children need to learn such process. At three to five months of age, infants produce more than a third of their turns in overlap (i.e., simultaneous speech) with their mothers’ and take a long time to respond (i.e., they show long between‐speaker pauses) (Bateson 1975). As children grow, their vocalization frequency in turn‐taking increases, while their simultaneous speech and between‐speaker pause durations decrease (Bateson 1975; Gratier et al. 2015; Hilbrink et al. 2015). Between‐speaker pauses appear to increase in duration again between nine and 12 months of age, when children face the challenge of using their emerging language skills within the given interactional timing (Hilbrink et al. 2015; Smith and McMurray 2018).

The parental language model that children are exposed to during early interactions shapes their language and communication learning processes (Hoff and Naigles 2002; Huttenlocher et al. 2010). Children who receive early exposure to a richer parental input in terms of conversational turns show more advanced expressive language skills (Gilkerson et al. 2017; Romeo et al. 2018). Moreover, the timing with which parental input is delivered is considered a crucial component of the linguistic model provided to children. In particular, the timing of caregivers’ responses has been shown to support preverbal development and early vocabulary acquisition (Gros‐Louis et al. 2014; Smith et al. 2024), as well as children's learning of conversational coordination and turn‐taking (Smith et al. 2023; Smith and McMurray 2018). Moreover, recent research has shown developmental and dyadic effects on timing of vocal behaviours in mother–child interactions: both partners are found to respond more quickly and with more consistent timing as children's age increases and to attune to and influence each other's timing (mothers’ response latency duration characteristics predict those of their children and vice versa) (Kondaurova et al. 2019; Smith et al. 2023, Smith et al. 2024; Smith and McMurray 2018). These findings suggest that mothers and children are sensitive to the temporal characteristics of the interaction and that children become increasingly more skilled at coordinating their vocal behaviours with those of their conversational partner as they grow up.

While learning vocal coordination skills is already expected to be a challenge for children with NH, it certainly poses an initially greater challenge to children who have had limited or no access to early auditory input, as is the case for children who are born deaf or hard of hearing (DHH) and later receive a cochlear implant (CI).

### 1.2 Coordination in NH Mother‐CI Child Dyads

Cochlear implantation allows DHH children to gain access to sound and to acquire spoken input. In the first year after they receive a CI, DHH children typically follow a trajectory of word acquisition that resembles that of children with NH (Li et al. 2020). Nonetheless, their early deprivation of, or limited access to, auditory input creates an early disadvantage in their language learning process that is reflected in their reduced vocabulary size and word productions compared to those of their age‐matched peers with NH (Kondaurova et al. 2020; Lund 2016; Majorano et al. 2020; Nittrouer et al. 2018).

The language development process of children with CIs has been shown to be heavily influenced by the quantity and quality of input that parents provide in early interactions (Curtin et al. 2021; DesJardin and Eisenberg 2007; Holzinger et al. 2020; Majorano et al. 2018; Persici et al. 2022; Quittner et al. 2013). As for their vocal behaviour in mother–child conversation specifically, we know from previous work that children with CIs show significantly increased vocalization frequency four to six months after implantation compared to before implantation (Fagan 2014; Fagan et al. 2014) and decreased simultaneous speech 12 months after implantation compared to three months after surgery (Kondaurova et al. 2019), suggesting a beneficial effect of CI use. In terms of timing, previous work has shown that DHH children wearing CIs or hearing aids are sensitive to the temporal dynamics of the interaction and adapt their vocal timing to that of their mothers (at least to some degree; see Smith and McMurray 2018). In addition, they undergo developmental changes, as their response latencies become shorter and less variable in duration as their age increases (Smith and McMurray 2018).

Mixed results have been found in recent work when the vocal behaviour of DHH children in interaction with their NH mothers is compared to that of children with NH. A few longitudinal studies have shown no effect of hearing group on the number of turns that children engage in (VanDam et al. 2012; Vanormelingen et al. 2016); others have shown early differences in the proportion of children's responses out of all the vocalizations that they produce which become not significant by 12 months after implantation (Kondaurova et al. 2019). In children with CIs tested three and 12 months post‐CI, generally longer between‐speaker pauses (i.e., their response latencies) than those of age‐matched children with NH have been found (Kondaurova et al. 2019). The between‐speaker pauses produced by children with CIs also appear to be longer than their within‐speaker pauses, whereas children with NH demonstrate the opposite pattern, similarly to adults (Kondaurova et al. 2019). These results have been interpreted as suggesting that hearing impairment affects the timing of turn‐taking. Moreover, a study has found that mother–child dyads with NH are tightly coupled in how quickly and variably they respond to each other, whereas mothers and children with CIs (i.e., mixed‐hearing dyads) show less temporal matching (Smith and McMurray 2018). As for simultaneous speech, previous work has provided evidence of greater proportions in mixed‐hearing dyads than in hearing dyads three months after implantation and similar proportions six and 12 months post‐CI (Fagan et al. 2014; Kondaurova et al. 2019). However, it is unclear from these studies whether this is also valid for children. Fagan et al. (2014) examined the simultaneous speech produced by mothers and Kondaurova et al. (2019) considered the total proportion of overlapping speech found in the dyad. Mothers and children may overlap their speech for different reasons: children may produce simultaneous speech because of their insufficiently developed timing skills, whereas mothers are expected to be skilled at coordinating their vocal behaviours in time and may produce more simultaneous speech in an attempt to re‐direct the child's attention.

When mothers of children with CIs have been observed, studies have found that they adapt their vocal behaviour to their child's age and vocal behaviour: mothers’ between‐speaker pauses become increasingly faster and less variable as children grow up and are predicted by their children's between‐speaker pause duration characteristics (Smith and McMurray 2018). The hearing status of their children also appears to have an effect: mothers of children with CIs produce more simultaneous speech before implantation than six months after surgery (Fagan et al. 2014). Mixed results are found when mothers of children with CIs are compared to those of children with NH: some studies have shown no significant differences in the number of turns that children with CIs and NH are exposed to (Vanormelingen et al. 2016) and no significant differences in mothers’ between‐speaker pause durations (Smith and McMurray 2018); others have shown fewer maternal responses in mixed‐hearing dyads in general (Kondaurova et al. 2019) and shorter between‐speaker pauses in the earlier stages after implantation (three months but not 12 months post‐CI) (Kondaurova et al. 2019).

Although recent research has significantly advanced our understanding of mother–child coordination in mixed‐hearing dyads, some findings remain inconclusive and are based on a relatively small number of studies, in some cases with limited sample sizes (e.g., eight DHH children in Smith and McMurray 2018), and/or short interaction durations (e.g., 5–7 min in Kondaurova et al. 2019), and almost all focusing on English‐speaking dyads.

Previous research has shown that some core principles of conversational turn‐taking, such as the tendency to minimize gaps and avoid extended overlaps, are remarkably robust across languages and cultures (Stivers et al. 2009). However, other aspects of turn‐taking, and in particular the fine‐grained timing of response latencies, appear to be culture‐specific. Cross‐linguistic evidence from adult interactions suggests that different languages are associated with shorter or longer response latencies, potentially reflecting culturally shaped norms regarding interactional pace and conversational rhythm (Stivers et al. 2009). Moreover, speakers from different language contexts vary in their tolerance of simultaneous speech: according to ethnographic reports, Italian speakers are more tolerant of simultaneous speech (Anolli et al. 2005) and may thus engage more frequently in these communicative behaviours compared to English speakers.

Finally, the specific characteristics of the language under study may also contribute to shaping conversational timing. Italian is traditionally characterized as a syllable‐timed language, in which syllables tend to have relatively similar durations and there is less reduction of unstressed vowels compared with stress‐timed languages such as English (Dauer 1983). These rhythmic properties may influence the temporal organization of speech and, in turn, the fine‐grained timing of turn‐taking in interaction.

It is important to note that the present study does not aim to provide a direct cross‐linguistic comparison between Italian and English. Rather, by focusing on Italian‐speaking caregiver‐child dyads, it seeks to examine whether the previously reported developmental and dyadic effects in caregiver‐child interaction (mostly documented in English‐speaking samples) generalize to other linguistic and cultural contexts.

### 1.3 Individual Factors Affecting Dyadic Timing of Vocal Behaviour

Another question of interest is whether dyadic timing is affected by individual characteristics. Previous work on hearing dyads has found that mothers at a higher risk of depression show slower response latencies in interaction with their children (Smith et al. 2023). In the present work, we asked whether timing at the dyad level could be positively influenced by higher levels of music engagement.

Musical activities are pervasive across cultures and frequently used in the early interactions of parents and children under the age of six years (Ilari 2021; Mehr 2014). These activities typically include singing, dancing, passive exposure to music, or playing musical games, and are mostly held in the home environment (Politimou et al. 2018). Research using questionnaires to investigate home music engagement has shown positive effects of engagement on the language development of children with NH (Franco et al. 2022) and CIs (Torppa et al. 2020; cf. Persici et al. 2024). Importantly, studies have also shown that participation in musical activities positively affects parents’ verbal responsiveness (Smith and Kong 2024) and the temporal coordination abilities of primary school DHH children in verbal interactions with an unfamiliar partner (Hidalgo et al. 2017, Hidalgo et al. 2019). Finally, a study in hearing adults by Wynn et al. (2022) has shown that individuals with more accurate rhythm perception skills—which typically correlate with amount of musical training (Müllensiefen et al. 2014)—tend to synchronize their vocal behaviour to that of the other speaker more effectively, that is, they show greater entrainment to the speaking rate of their interlocutor, with beneficial effects on conversational quality. It has been argued that this is possible because individuals who have enhanced perceptual (rhythmic) abilities also have a greater ability to extract rhythmic information from the speech signal of their conversational partner and to integrate it into their own speech pattern (Wynn et al. 2022).

Thus, in previous work musical activities have been linked to enhanced coordination between conversational partners who were either both adults, or a DHH child and an unfamiliar adult. In the present study, we explore the hypothesis that similar effects of musical activities could be found on coordination in dyads of mothers with NH and children with CIs who are in the preschool years. More specifically, we hypothesized that dyads with greater home music engagement would show enhanced vocal behaviour timing skills in interaction after implantation.

Although hearing impairment and musical experience are often examined as independent factors, they can also be conceptualized within a broader framework of environmental deprivation and enrichment. Hearing impairment (as well as CIs) limits access to fine‐grained acoustic and temporal cues that support the development of conversational timing, whereas engagement in musical activities has been shown to enhance temporal prediction, rhythmic processing, and auditory–motor coordination. Examining these factors jointly therefore allows us to test the extent to which early turn‐taking dynamics are sensitive to reductions and enhancements in the quality of the auditory and temporal environment.

### 1.4 The Present Study

The present study investigates mother–child coordination in mixed‐hearing and hearing dyads at two time points, corresponding to before children received their CI (T1) and one year after implantation (T2) for the group of hearing mothers and children with CIs (henceforth, CI group), and explores the hypothesis that vocal behaviour in early exchanges is influenced by amount of home music engagement. Home music engagement data for the same participants were previously reported in Persici, Santangelo, et al. (2024). The current study presents novel data on mothers’ and children's vocal behaviour in interaction and novel analyses on their relationship with these previously reported scores.

The aims of the present study are three:

***Aim 1)*** to investigate whether turn‐taking and mother–child coordination differs across hearing groups and sessions. The following behaviours were examined: relative frequency of temporally contingent responses (out of all vocalizations), relative frequency of simultaneous speech (out of all vocalizations), timing of within‐ and between‐speaker pauses.

We expected to find fewer temporally contingent responses for children with CIs at T1 than at T2, and in general compared to children with NH, due to their weaker language skills (Persici, Castelletti et al. 2024). As a result of their limited language productions and based on previous work (e.g., Kondaurova et al. 2019), we also expected mothers in the CI group to produce fewer temporally contingent responses compared to mothers in the NH group.

Based on Fagan et al. (2014), we expected to find more frequent simultaneous speech for mothers in the CI group than for those in the NH group at T1, and that this difference was reduced and no longer significant at T2. Because we expected children with CIs to have less efficient vocal coordination abilities compared to children with NH, we hypothesized to find a higher proportion of simultaneous speech relative to their total vocalizations in the CI group than in the NH group at both sessions.

As for pauses, we expected (i) all mothers to show longer within‐ than between‐speaker pauses and (ii) mothers in the CI group to produce longer and more variable between‐speaker pauses than mothers in the NH group at T1 but not at T2 (Kondaurova et al. 2019). We anticipated that only children with NH would show a similar pattern to that of their mothers and that they would show shorter and less variable pauses than children with CIs in general (Kondaurova et al. 2019).

***Aim 2)*** to investigate whether there are reciprocal effects between mother and child at T1 and T2. Following previous work (Kondaurova et al. 2019; Smith and McMurray 2018), we examined whether variance in children's vocal behaviour was explained by that of their mothers, beyond effects of hearing group and session. Then, we investigated the possibility of reciprocal effects in the other direction. However, while previous work applied this methodology to between‐speaker pauses only, in the present work we investigated the presence of reciprocal effects on all the vocal behaviours listed above (see Aim 1). We expected to replicate previous findings showing significant effects of children's (between‐speaker) pause durations on mothers’ and no significant effects of mothers on children (Kondaurova et al. 2019). Moreover, we hypothesized to find similar results for all the other vocal behaviours investigated here.

***Aim 3)*** to explore, in the sub‐group of children with CIs and their mothers at T2, whether vocal behaviour at the dyad‐level is positively affected by amount of home music engagement. T1 was not taken into consideration, because before implantation children with CIs had limited or no access to auditory input. We expected that those mixed‐hearing dyads who had greater levels of music engagement in the home environment at T2 would show enhanced timing skills in conversation in the same session.

## 2 Methods

### 2.1 Participants

Thirty‐four dyads participated in the study. Seventeen were dyads composed of mothers with NH and children with CIs (mean age at T1 = 17.12 months, *SD* = 7.76; mean age at T2 = 30.52 months, *SD* = 7.53) (henceforth, CI group). These dyads were recruited at the ‘Guglielmo da Saliceto’ Hospital before children received their CI and were enrolled in a larger study on language development (Majorano, Brondino, et al. 2020; 2021; Persici et al. 2022; Persici, Castelletti et al. 2024; Persici, Santangelo et al. 2024). The criteria for inclusion in the present study were the following: for children, a diagnosis of severe to profound bilateral sensorineural deafness, planned CI implantation by 36 months of age (associated with better language outcomes; e.g., Connor et al. 2006), absence of diagnosed sensorimotor or developmental disorders and cognitive disabilities, parents with NH, no exposure to other languages including sign, and weekly participation in an auditory‐verbal therapy rehabilitation program; mothers had to have reported bilateral NH and be monolingual speakers of Italian. Both partners had to have participated in both sessions. Children had a mean PTA of 100.88 dB/HL (*SD* = 5.37) at T1, a mean age at diagnosis of 7.24 months (*SD* = 8.59), and a mean age at implantation of 17.12 months (*SD* = 7.76). Children's vocabulary abilities, measured using the Italian version of the ‘Words and Gestures’ short form of the MacArthur–Bates Communicative Development Inventory (MB‐CDI) (Caselli et al. 2015) at T1 and T2 and the number of tokens and types produced by children and their mothers during the interactions were already reported in Persici, Castelletti, et al. (2024). In the CI group, children's mean receptive vocabulary raw score at T1 was 10.42 (*SD* = 11.77), their mean expressive vocabulary scores were 0.59 (*SD* = 1.42) at T1 and 26.24 (*SD* = 25.85) at T2. Children produced an average of 0.25 tokens (*SD* = 0.80) and 0.07 types (*SD* = 0.16) per minute at T1 and an average of 3.43 tokens (*SD* = 2.57) and 1.96 types (*SD* = 1.92) per minute at T2. Mothers in the CI group produced an average of 43.73 tokens (*SD* = 18.89) and 10.78 types (*SD* = 4.43) per minute at T1 and an average of 54.93 tokens (*SD* = 19.10) and 13.88 (*SD* = 3.83) per minute at T2. For more details on these measures, please see Persici, Castelletti et al. (2024).

A second group of dyads was constituted by mothers and children with NH (*n* = 17; mean age at T1 = 17.06 months, *SD* = 7.36; mean age at T2 = 30.9 months, *SD* = 8.8) (henceforth NH group). This group was recruited from early education centres in northern Italy and enrolled in the study if children had reported bilateral hearing and no diagnosis of sensorimotor or developmental disorders and/or cognitive disabilities. The criteria for mothers were the same as for the CI group. Children with NH were matched with children with CIs based on chronological age at the end of study (*t*(16) = 0.324, *p* = 0.750). See Table 1 for individual characteristics.

**TABLE 1:** Child participant characteristics.

Subject	Sex	Aetiology	Age at diagnosis (months)	Age at implantation (months)	PTA (dB/HL) Unaided CI side	Age at T1 (months)
CI1	M	Genetic (Cx26)	2	13	100	13
CI2	F	Genetic (Cx26)	2	10	105	10
CI3	M	Genetic (Cx26)	10	18	100	18
CI4	F	Unknown	24	30	100	30
CI5	F	Unknown	11	25	95	25
CI6	F	Genetic (Cx26)	2	11	100	11
CI7	F	Unknown	28	33	105	33
CI8	F	Cytomegalovirus	2	13	90	13
CI9	M	Genetic (Cx26)	2	11	105	11
CI10	M	Unknown	6	15	100	15
CI11	M	Cytomegalovirus	2	12	105	12
CI12	F	Unknown	3	11	90	11
CI13	M	Unknown	2	15	100	15
CI14	M	Unknown	2	11	105	10
CI15	F	Genetic (Cx26)	20	31	110	31
CI16	M	Genetic (Cx26)	3	19	105	19
CI17	M	Genetic (Cx26)	2	13	100	13
NH1	M	—	—	—	—	13
NH2	F	—	—	—	—	10
NH3	M	—	—	—	—	19
NH4	F	—	—	—	—	29
NH5	F	—	—	—	—	24
NH6	F	—	—	—	—	12
NH7	F	—	—	—	—	32
NH8	F	—	—	—	—	14
NH9	M	—	—	—	—	11
NH10	M	—	—	—	—	15
NH11	M	—	—	—	—	11
NH12	F	—	—	—	—	11
NH13	F	—	—	—	—	15
NH14	M	—	—	—	—	11
NH15	F	—	—	—	—	30
NH16	M	—	—	—	—	20
NH17	M	—	—	—	—	13

*Note*: T1 = before implantation for the children with CIs; T2 = 1 year post‐CI activation.

As reported in Persici, Castelletti, et al. (2024), in the NH group, children showed an average receptive vocabulary raw score of 43 (*SD* = 32.44) at T1 and an average expressive vocabulary of 22.06 (*SD* = 35.01) at T1 and of 78.06 (*SD* = 28.17) at T2. Children produced an average of 1.60 tokens (*SD* = 2.80) and 0.71 types (*SD* = 1.36) per minute at T1 and an average of 7.17 tokens (*SD* = 3.48) and 3.97 types (*SD* = 2.26) at T2. Their mothers produced an average of 52.23 tokens (*SD* = 17.55) and 14.54 types (*SD* = 4.65) per minute at T1 and an average of 58.32 tokens (*SD* = 13.38) and 17.29 (*SD* = 3.68) per minute at T2.

The study was approved by the Ethics Committee of the University of Verona (protocol number 2017/46268). Mothers signed an informed consent form.

### 2.2 Materials

#### 2.2.1 Mother–Child Interaction

In each session, dyads were observed individually during semi‐structured play for approximately 20 min. Both sessions were led by a doctoral student in Developmental Psychology following an adaptation of the Assessing Linguistic Behaviour protocol (Olswang et al. 1987) and were videorecorded using a video camera with a built‐in 5.1‐channel surround microphone. Each dyad received four sets of age‐appropriate toys, introduced sequentially every 5 min in a predetermined order, with the aim to elicit as many diverse words as possible. Mothers were instructed to use all toys and to play with their children as they usually did at home.

#### 2.2.2 Children's Home Music Engagement

Amount of music engagement at home for mixed‐hearing dyads was evaluated using the parent report Music@Home—Infant Questionnaire (Politimou et al. 2018; adaptation in Italian by Franco and Boem, 2018). The Infant questionnaire was designed to assess the home music environment of preverbal children and was thus deemed suitable for our sample as well, despite our participants being chronologically older than those in Politimou et al. (2018). The questionnaire comprises four subscales: (1) parental beliefs (four items), (2) child engagement with music (six items), (3) parent initiation of singing (five items), and (4) parent initiation of music making (three items). Parents were required to indicate their level of agreement with a given statement using a 7‐point Likert scale (from “completely disagree” to “completely agree”). The total score was calculated by combining items from each subscale (see Persici, Santangelo, et al. 2024, for more information).

### 2.3 Procedure

The audio track of the video recordings was extracted and imported into Praat (Boersma and Weenink 2018). Mothers’ and children's speech was coded in two separate tiers. The beginning and end of each vocal behaviour was defined through careful listening and observation of the waveform and spectrogram. Following previous work (Gratier et al. 2015; Kondaurova et al. 2019), vocalizations were defined as vocal sounds that were produced by a speaker either continuously or including a within‐speaker pause with a duration shorter than 300 ms. If the duration of the within‐speaker pause included was longer than 300 ms, two separate vocalizations were coded. All vegetative sounds were labelled and later excluded from analysis.

Vocalizations were further coded as *temporally contingent responses* if they occurred within a latency of 5000 ms from the other speaker's previous vocalization (as in Smith et al. 2023; Smith and McMurray 2018).

Vocalizations that were produced in overlap with the other speaker's vocalizations were coded as either *interruptive* (ISS) or *non‐interruptive simultaneous speech* (NSS), depending on whether or not the person who intervened ended up taking the turn (Jaffe et al. 2001).

Pauses were coded as *within‐speaker pauses* if they occurred between two vocalizations produced by the same speaker and had a duration longer than 300 ms. Pauses between two vocalizations of two different speakers were coded as *between‐speaker pauses*. For example, if a vocalization produced by the mother was followed by a pause and by a vocalization by the child, the pause was labelled as the children's between‐speaker pause. Note that these pauses correspond to what in previous work has also been called ‘response latency’ (e.g., Smith and McMurray 2018). For both types of pauses, all pauses with a duration above 5 s were discarded, as in previous work (Smith et al. 2023; Smith and McMurray 2018). This action led to the removal of 125 between‐speaker pauses (2.3% of the total between‐speaker pauses) for mothers and of 151 between‐speaker pauses (2.7%) for children. No within‐speaker pauses were removed as a consequence of this action.

The Music@Home questionnaire was filled out by mothers of children with CIs with reference to T1 and gained retrospectively after the end of the study. Note that it was not possible to obtain the same data from the parents of children with NH.

#### 2.3.1 Inter‐Coder Reliability

The data was coded by one master and four undergraduate students trained by the first author. Twenty percent of the material (12 recordings in total) was double coded by the first author. Inter‐coder reliability for the number of labelled behaviours for mothers and children, separately, was checked by calculating the intraclass correlation coefficient (ICC) using two‐way random effect models and “single rater” unit (*psych* package; Revelle 2024) in R (R Core Team 2024). The ICC for mothers ranged between 0.928 and 0.992 (vocalizations: 0.990; ISS: 0.983; NSS: 0.928; between‐speaker pauses: 0.992; within‐speaker pauses: 0.990). The ICC for children ranged between 0.955 and 0.991 (vocalizations: 0.964; ISS: 0.979; NSS: 0.955; between‐speaker pauses: 0.991; within‐speaker pauses: 0.980). As in previous work (Hilbrink et al. 2015; Smith et al. 2024), the timing of vocalizations and pauses was considered identical if the difference between their start or end times was 80 ms or shorter. Mean inter‐coder agreement for onsets and offsets was 91% and 80%, respectively.

#### 2.3.2 Data Analysis

Each coded file was then extracted from Praat and imported in R for data analysis. Relative frequency of temporally contingent responses and simultaneous speech for each speaker was calculated as the proportion of temporally contingent responses or simultaneous speech out of all the vocalizations that the same speaker produced (Kondaurova et al. 2019). Timing of vocal behaviours was investigated using two measures: mean duration and duration variability (Smith and McMurray 2018). Mean duration was calculated as the difference in seconds between the offset and onset of each labelled vocal behaviour. Duration variability was calculated as the SD of the duration of each labelled vocal behaviour.

Potential differences across groups and sessions in relative frequency and timing of mothers’ and children's vocal behaviour (*Aim 1*) were investigated using a series of Linear Mixed‐effects Models (LMMs) with Group (NH, CI), Session (T1, T2), and their interaction as fixed predictors, and with Subject as random intercept. The *t*‐statistic and the Satterthwaite approximation for degrees of freedom were used to assess significance of the fixed effects (lmerTest package; Kuznetsova et al. 2017). For simultaneous speech and pauses, Type (ISS, NSS for simultaneous speech; within, between for pauses) and its interactions with the other predictors were also included as fixed predictors. Including Type as a fixed factor in the same model allowed us to directly assess differences between types and to test whether they showed systematically different patterns across groups and sessions, through interactions with the relevant predictors. Moreover, it also allowed us to account for shared variance among predictors and may increase statistical power compared to fitting separate models.

To investigate reciprocal effects between mother and child (*Aim 2*) above the effects of group and session (and type, in the case of simultaneous speech and pauses), we followed the same approach used by Smith and colleagues (Kondaurova et al. 2019; Smith et al. 2023; Smith and McMurray 2018). We first build a series of baseline LMMs with Group and Session (and Type) as fixed predictors and Subject as random effect. Then, we compared these baseline models with models that additionally included the other speaker's behaviour, using Chi square tests. For example, for children's response relative frequency, we compared the baseline model with Group and Session as fixed predictors with a model that additionally included mothers’ proportion of responses as fixed predictor. Finding that the more complex model provides a better fit to the data would suggest that children adapt their vocal behaviour to that of their mothers at the dyad level.

Finally, we explored the hypothesis that dyad‐specific vocal behaviours at T2 would be affected by amount of home music engagement (*Aim 3*). Mothers’ and children's vocal behaviours at T2 were combined into the same dataset. A set of LMMs were built on the following vocal behaviour characteristics at T2 as dependent variables: proportion of responses, proportion of simultaneous speech (ISS and NSS), (within and between‐speaker) pause mean duration, and (within and between‐speaker) pause duration variability. In the models on simultaneous speech relative frequency and pause duration, Type was included as additional predictor. By including this predictor, which was expected to soak up some of the variance, we hoped to get a more accurate estimate of the effect of home music engagement. In all models, Dyad was included as random intercept.

## 3 Results

Relative frequency of temporally contingent responses and of interruptive and non‐interruptive simultaneous speech (proportion values), and duration means and SDs for within‐ and between‐speaker pauses are reported in Table 2 by group, session, and speaker.

**TABLE 2:** Mean relative frequency of responses and simultaneous speech (proportion values) and mean duration characteristics of within‐ and between‐speaker pauses by group, session, and speaker. SDs are in parentheses.

	CI group				NH group			
	T1		T2		T1		T2	
	Mother	Child	Mother	Child	Mother	Child	Mother	Child
Proportion of temporally contingent responses (0–1)	0.15 (0.11)	0.50 (0.12)	0.28 (0.12)	0.60 (0.10)	0.19 (0.13)	0.56 (0.12)	0.39 (0.16)	0.62 (0.07)
Proportion of ISS (0–1)	0.07 (0.05)	0.26 (0.13)	0.09 (0.05)	0.19 (0.10)	0.06 (0.04)	0.17 (0.09)	0.12 (0.06)	0.13 (0.07)
Proportion of NSS (0–1)	0.04 (0.06)	0.18 (0.12)	0.05 (0.04)	0.09 (0.05)	0.02 (0.01)	0.14 (0.08)	0.02 (0.01)	0.07 (0.04)
Duration of within‐speaker pauses (seconds)	1.35 (0.27)	0.99 (0.46)	1.27 (0.19)	1.02 (0.27)	1.42 (0.22)	1.15 (0.36)	1.33 (0.21)	1.04 (0.26)
Duration of between‐speaker pauses (seconds)	0.73 (0.31)	0.92 (0.28)	0.62 (0.17)	0.91 (0.18)	0.74 (0.22)	1.06 (0.29)	0.60 (0.11)	0.93 (0.27)

Abbreviations: ISS = interruptive simultaneous speech; NSS = non‐interruptive simultaneous speech.

### 3.1 Aim 1: Differences Across Groups and Sessions in Relative Frequency of Temporally Contingent Responses and Simultaneous Speech, and in Timing of Pauses

Table 3 shows the highest‐level significant (or marginally significant) effects or interactions in the models on relative frequency, mean duration, and duration variability. The results are reported below by speaker and variable. All effects and interactions are reported in Table S1 in the Supplementary materials. The full model summaries are reported in Table S2 in the Supplementary materials.

**TABLE 3:** Highest‐level significant or marginally significant effects or interactions in the models on mothers’ and children's relative frequency or timing of vocal behaviours in conversation.

		Effect or interaction	*F*	*df*	*p*
Mothers					
Temporally contingent responses	Proportion	Group	5.104	1, 32.209	0.031
		Session	30.309	1, 32.099	< 0.001
Simultaneous speech (ISS, NSS)	Proportion	Group × Type	10.596	1, 88.071	0.002
		Session × Type	8.049	1, 86.209	0.006
Pauses (within, between)	Mean duration	Session	10.346	1, 94.026	0.002
		Type	390.625	1, 92.705	< 0.001
	Duration variability (SD)	Session	6.487	1, 94.924	0.013
		Type	32.124	1, 93.467	< 0.001
Children					
Temporally contingent responses	Proportion	Session	16.464	1, 31.642	< 0.001
Simultaneous speech (ISS, NSS)	Proportion	Group	8.006	1, 30.791	0.008
		Session	21.131	1, 92.837	< 0.001
		Type	23.381	1, 91.498	< 0.001
Pauses (within, between)	Mean duration	Type	3.522	1, 94.345	0.064
	Duration variability (SD)	Type	11.673	1, 92.259	< 0.001

Abbreviations: ISS = interruptive simultaneous speech; NSS = non‐interruptive simultaneous speech.

Previous research has shown that age at diagnosis (Yoshinaga‐Itano et al. 1998) and age at implantation (Nicholas and Geers 2007) affect the spoken language development of children with CIs. For the purpose of investigating whether age at diagnosis and implantation were associated with children's relative frequency of temporally contingent responses, simultaneous speech, and pause timing, we ran correlation analyses between age at diagnosis and our variables of interest at T1 and T2, and between age at implantation and our variables at T2. Results showed no significant associations, with the exception of a negative correlation between age at implantation and children's mean duration of within‐speaker pauses at T2 (*r* = −0.54, *p* = 0.032).

#### 3.1.1 Mothers

##### 3.1.1.1 Temporally Contingent Responses

The model on the relative frequency of the subgroup of vocalizations that were *temporally contingent responses* to the child's previous vocalization showed main effects of Group and Session. Mothers produced twice as many temporally contingent responses at T2 (*M* = 0.34) than at T1 (*M* = 0.17) (*t*(31.7) = −5.502, *p *< 0.001), and mothers in the NH group produced more responses than mothers in the CI group (*M* = 0.29 vs. 0.21; *t*(31.8) = 2.258, *p* = 0.031).

##### 3.1.1.2 Simultaneous Speech

We found significant interactions between Group and Type and between Session and Type. To unpack the Group × Type interaction, follow‐up comparisons showed that, within both groups, ISS was significantly more frequent than NSS (CI group: *M* = 0.08 vs. 0.05; *t*(88.4) = 3.526, *p *< 0.001; NH group: *M* = 0.09 vs. 0.02; *t*(89.0) = 8.075, *p *< 0.001). Comparing groups, NSS was more frequent in the CI group than in the NH group (*M* = 0.05 vs. 0.02; approximately 2.5 times higher; *t*(56.7) = 2.252, *p* = 0.028).

Regarding the Session × Type interaction, ISS was twice as frequent as NSS at T1 (*M* = 0.06 vs. 0.03; *t*(88.5) = 3.784, *p *< 0.001) and more than three times as frequent at T2 (*M* = 0.10 vs. 0.03; *t*(87.2) = 7.946, *p *< 0.001). Overall, ISS was significantly more frequent at T2 than at T1 (*M* = 0.09 vs. 0.06; *t*(86.7) = 4.648, *p *< 0.001).

##### 3.1.1.3 Pauses

Descriptive statistics showed that between‐speaker pauses were shorter at T2 than at T1 for both the NH group (*M* = 0.60 vs. 0.74) and the CI group (*M* = 0.62 vs. 0.73). Given the approximately 12‐month interval between sessions, this corresponds to an average reduction of about 12 ms/month in the NH group and 9 ms/month in the CI group. Between‐speaker pause variability was lower at T2 than at T1 in both groups (NH group: *M* = 0.73 vs. 0.86; average decrease of 11 ms/month; CI group: 0.70 vs. 0.78; average decrease of 7 ms/month).

Within‐speaker pauses were also shorter at T2 than at T1 for both the NH group (*M* = 1.33 vs. 1.42) and the CI group (*M* = 1.27 vs. 1.35), corresponding to an average reduction of 7 ms/month in both groups. Within‐speaker pause variability was lower at T2 than at T1 in both groups (NH group: *M* = 0.94 vs. 1.02; average decrease of 7 ms/month; CI group: *M* = 0.92 vs. 1.00; average decrease of 7 ms/month).

The results of the model showed significant main effects of Session and Type for both mean duration and duration variability, with mothers’ pauses being significantly longer and more variable at T1 than at T2 (mean duration: *M* = 1.06 vs. 0.95; *t*(95.4) = 3.215, *p* = 0.002; variability: *M* = 0.91 vs. 0.82; *t*(95.6) = 2.546, *p* = 0.013). Finally, within‐speaker pauses were significantly longer and more variable than between‐speaker pauses (mean duration: *M* = 1.34 vs. 0.67, *t*(94.1) = 19.764, *p *< 0.001; variability: *M* = 0.97 vs. 0.77, *t*(94.2) = 5.668, *p *< 0.001).

### 3.2 Children

#### 3.2.1 Temporally Contingent Responses

As reported in Table 2, analyses showed a main effect of Session for proportion of responses, with responses being more frequent at T2 than at T1 (*M* = 0.61 vs. 0.53; *t*(31.5) = 4.055, *p *< 0.001).

#### 3.2.2 Simultaneous Speech

Tests only showed significant main effects of Group, Session, and Type on proportion of simultaneous speech. Overall, the CI group produced simultaneous speech more frequently than the NH group (*M* = 0.18 vs. 0.13; *t*(31.8) = 2.829, *p* = 0.008). Children generally produced simultaneous speech more frequently at T1 than at T2 (*M* = 0.19 vs. 0.12; *t*(93.9) = 4.594, *p *< 0.001). Finally, ISS was generally more frequent than NSS (*M* = 0.19 vs. 0.12; *t*(92.6) = 4.835, *p *< 0.001).

#### 3.2.3 Pauses

Descriptive statistics showed that between‐speaker pauses were shorter at T2 than at T1 for the NH group (*M* = 0.93 vs. 1.06), corresponding to an average reduction of 11 ms/month. Mean duration was almost identical for the CI group (*M* = 0.92 vs. 0.91). Duration variability was lower at T2 than at T1 in the NH group (*M* = 0.91 vs. 1.04; average decrease of 11 ms/month) and quite stable in the CI group (*M* = 0.94 at T2 vs. 0.92 at T1; average increase of 2 ms/month).

Similar results were found for within‐speaker pause duration (NH group: *M* = 1.04 at T2 vs. 1.15 at T1; average reduction of 9 ms/month; CI group: *M* = 1.02 vs. 0.99; average reduction of 2.5 ms/month) and variability (NH group: *M* = 0.78 vs. 0.84; average decrease of 5 ms/month; CI group: *M* = 0.75 at both sessions).

Tests showed a marginally significant effect of Type on mean duration, with marginally longer within‐ than between‐speaker pauses in general (*M* = 1.05 vs. 0.95; *t*(93.6) = 1.877, *p* = 0.064).

As for variability, we found a significant main effect of Type, with between‐speaker pauses being generally more variable in duration than within‐speaker pauses (*M* = 0.95 vs. 0.78; *t*(91.4) = 3.415, *p* = 0.001).

#### 3.2.4 Aim 2: To Investigate Whether Mothers and Children Affect Their Vocal Behaviours Reciprocally

First, we investigated whether children's vocal behaviours were predicted by their mothers’, over and above the effects of group and session (or group, session, and type for simultaneous speech and pauses). Then, we examined whether mothers’ vocal behaviours were predicted by their children's, over and above the effects of group and session or group, session, and type.

##### 3.2.4.1 Effects of Mothers’ Vocal Behaviour on Their Children's

##### 3.2.4.2 Temporally Contingent Responses

The model including mothers’ proportion of responses on children's proportion of responses did not outperform the base model with Group and Session (*p* = 0.392).

##### 3.2.4.3 Simultaneous Speech

The model on children's proportion of simultaneous speech including mothers’ proportion of simultaneous speech did not outperform the base model (*p* = 0.670).

##### 3.2.4.4 Pauses

The model on children's pause mean duration including mothers’ pause mean duration outperformed the base model with Group, Session, and Type (*χ* ^2^_(1)_ = 4.256, *p* = 0.039). The effect of the added predictor was positive and significant (*b* = 0.269, *S.E*. = 0.126, *p* = 0.035). The model on children's pause duration variability including mothers’ had a significantly better fit to the data than the base model with Group, Session, and Type (*χ* ^2^_(1)_ = 8.047, *p* = 0.005). The effect of mothers’ pause duration variability was positive and significant (*b* = 0.392, *S.E*. = 0.129, *p* = 0.003).

#### 3.2.5 Effects of Children's Vocal Behaviour on Their Mothers’

##### 3.2.5.1 Temporally Contingent Responses

The model including children's proportion of responses on mothers’ proportion of responses did not outperform the base model with Group and Session (*p* = 0.309).

##### 3.2.5.2 Simultaneous Speech

Analyses revealed that the model including children's proportion of simultaneous speech did not outperform the base model not including it (*p* = 0.834).

##### 3.2.5.3 Pauses

The model on mothers’ pause mean duration including children's pause mean duration did not outperform the base model with Group, Session, and Type (*p* = 0.145).

The model on mothers’ pause duration variability including children's pause duration variability outperformed the base models with Group, Session, and Type (*χ* ^2^_(1)_ = 7.840, *p* = 0.005), and the effect of the added predictor was significant (*b* = 0.191, *S.E*. = 0.063, *p* = 0.003).

### 3.3 Aim 3: In the CI Group, to Explore Whether Home Music Engagement at T2 Predicts Vocal Behaviour at the Dyad Level

Results showed no significant effect of the Music@Home total score at T2 on the dyads’ proportion of responses (*p* = 0.490) or of overlaps (*p* = 0.834) in the same session.

The model on dyads’ pause mean duration showed a significant and negative effect of the Music@Home score at T2 (*ß* = −0.304, *S.E*. = 0.114, *p* = 0.011). Similar results were found on pause duration variability (*ß* = −0.313, *S.E*. = 0.148, *p* = 0.041).

## 4 Discussion

The present study examined turn‐taking and mother–child coordination in two groups of children (with NH or CIs) matched for chronological age across two time points, corresponding to before implantation (T1) and one year after CI activation (T2) for the children with CIs. Moreover, we investigated whether there are reciprocal effects between mother and child at T1 and T2, and whether individual differences at the dyad level in mixed‐hearing dyads are to be attributed to individual differences in amount of home music engagement. Findings are discussed below by aim.

### 4.1 Group and Session Differences in Turn Taking and in Mother–Child Coordination

We found that both mothers and children responded to each other more frequently at T2 than at T1, independently of group. This suggests that interactions become richer in conversational turns as children grow older and gain more experience and skills, in line with previous work (Gratier et al. 2015; Hilbrink et al. 2015; Vanormelingen et al. 2016). In line with our hypothesis, we found an effect of children's hearing: mothers of children with CIs generally produced less frequent temporally contingent responses than mothers of children with NH across sessions. As reported in Table 3, the proportion of temporally contingent responses produced by children with CIs was also lower, but this between‐group difference did not reach statistical significance. These findings are in line with previous work showing fewer vocal turns in the interactions of mixed‐hearing dyads (Kondaurova et al. 2019; Lederberg and Mobley 1990).

In terms of simultaneous speech, we found that the more frequent type, namely ISS, was significantly more frequent in mothers’ speech at T2 than at T1, across groups. This finding may reflect a maternal tendency to redirect the conversation when children are older and more vocally active in the interaction (as evidenced by their relative increase of temporally contingent responses over time). In fact, in this specific type of situation, mothers joined in and persisted until they successfully took the turn. The other type of simultaneous speech, namely NSS, was produced more frequently by mothers in the CI group than by those in the NH group across sessions. In other words, mothers of children with CIs attempted more frequently to interrupt their children (and failed), both before implantation and one year later, compared to mothers of children with NH. This result has two key implications. First, it suggests that mothers of children with CIs had a more directive conversational style compared to mothers of children with NH, consistent with previous work on speakers of other languages (Adi‐Bensaid and Greenstein 2020; Fagan et al. 2014). Second, their lack of success in taking the turn may indicate that children with CIs were less receptive to their mothers’ interruptions than children with NH. This may depend on their limitations in acoustic processing and/or on a necessity to prioritize their own speech production and the formulation of their own utterances over monitoring their mother's speech, due to delayed language acquisition.

As for children, we found that simultaneous speech was generally more frequent at T1 than at T2. We interpret this result as suggesting that, as children gain more experience with conversational interactions, they become more able to coordinate their speech productions with those of their interlocutor, independently of whether they have NH. However, we also found that children with CIs produced more frequent simultaneous speech (especially ISS) compared to the NH group across sessions. This suggests that children with CIs may indeed have less efficient vocal coordination abilities compared to children with NH, perhaps due to having less experience with auditory input and managing perception and production. Moreover, their limitations in acoustic processing may reduce their ability to perceive subtle pitch cues that signal the end of a turn (Mehta and Oxenham 2017), increasing the likelihood that they start producing speech before their mothers’ turn is finished. The finding of this result across sessions is partially in contrast with Kondaurova et al. (2019), who found more simultaneous speech in mixed‐hearing dyads before implantation but similar levels across groups one year post‐CI. While our results may indicate that the children in our sample had not caught up with their peers in terms of coordination abilities one year post‐CI, it is also important to note that Kondaurova et al. (2019) did not distinguish between simultaneous speech initiated by mothers versus children. Their findings provide a broader perspective on interaction dynamics in mixed‐hearing dyads and are not necessarily in contrast with ours. Although analysing the total amount of overlapping speech provides information on the degree of coordination of the dyad, it does not tell us who is more responsible of these behaviours. The different results found for mothers’ and children's simultaneous speech productions in the present work suggest that future research should also investigate who initiates the overlapping productions.

In terms of pauses, we found that mothers’ were generally longer and more variable in duration at T1 than at T2, which confirms previous research showing that mothers tend to become more stable in their pause timing as children age and increase their contribution in the conversation (Smith et al. 2023; Smith and McMurray 2018). Analyses highlighted an average decrease in mothers’ between‐speaker pause (or response latency) duration of 12 ms/month in the NH group and of 9 ms/month in the CI group. The rate of decrease observed in the NH group closely matches that reported by Smith and McMurray (2018; 17 ms/month) and is identical to that reported by Smith et al. (2023), despite differences in sample characteristics and first language (English vs. Italian). The decrease in duration variability observed in the present work (−11 ms/month) was also consistent with previous research (−10 ms/month in Smith and McMurray 2018). This convergence across studies suggests that the developmental reduction in response latency duration and variability reflects a general property of caregiver–child interaction that is not tied to the specific rhythmic or prosodic properties of a given language. Rather, it likely indexes domain‐general changes in conversational coordination and temporal alignment between caregivers and children as communicative skills mature.

We also found that mothers produced longer within‐ than between‐speaker pauses, in line with our hypothesis and the typical timing of interactional pauses in adults (Kondaurova et al. 2019), and that within‐speaker pauses were also more variable in duration. This suggests that, when mothers responded to their children, they did so with more stable timing across turns. These patterns appeared to be similar across groups; in contrast with our hypothesis and Kondaurova et al. (2019), we did not find between‐group differences in mothers’ pause timing at T1.

Developmentally, children's between‐speaker pauses showed a clear reduction in terms of duration and variability in the NH group (approximately 11 ms/month), whereas no comparable developmental change was observed in the CI group. Again, the former result closely matches those reported in previous work on different populations (e.g., −12 ms/month in duration reported in Smith et al. 2023 and −15 ms/month in duration and −9 ms/month in variability reported in Smith and McMurray 2018). Nonetheless, no significant differences emerged in the models on children's pause timing between sessions and/or groups, potentially as a result of the small sample size.

Children generally showed marginally shorter and significantly more variable between‐speaker pauses than within‐speaker pauses. The former finding highlights an emerging tendency supporting our hypothesis and is in line with mothers' data; the latter suggests that children were more stable in their pauses between vocalizations within turn and comparatively less so when responding to their mothers, in sharp contrast with their mothers’ behaviour. Thus, children demonstrate to be in the process of learning the timing characteristics of the interaction, but not to be yet adult‐like in their response timing between turns. Interestingly, in the present work this was true for children across the two groups, in contrast with the results on between‐speaker pauses reported in Kondaurova et al. (2019). Taken together, these findings support the idea that timing skills in interaction follow a developmental trajectory (Hilbrink et al. 2015; Levinson and Torreira 2015). More research is needed to clarify whether children with CIs show a slower development.

### 4.2 Reciprocal Influences Between Mothers and Children

Our second aim was to investigate whether mothers’ and children's vocal behaviours influenced each other reciprocally. In contrast with our hypothesis, we found no reciprocal effects on relative frequency of temporally contingent responses and/or simultaneous speech. In other words, mothers were not more responsive or intrusive when their children were more responsive or intrusive, and vice versa. We speculate that mothers were relatively unaffected by their children's vocal behaviours because degree of responsiveness and frequency of simultaneous speech also depend on individual characteristics such as loquacity, stress levels, depressive symptoms, and cultural influences (Anolli et al. 2005; Field 1984; Huttenlocher et al. 2007; Ward and Lee 2020). Children may be relatively unaffected by their mothers’ frequency of temporally contingent responses and simultaneous speech at this stage, because their language and communicative abilities are still limited.

As for the timing of mothers’ and children's vocal behaviours, we found that mothers’ pause duration variability (but not their duration) was influenced by that of their children, above effects of group, session, and type of pause. Children's pause duration and variability were both predicted by their mothers’ pause characteristics, in line with previous work on English‐speaking dyads (Kondaurova et al. 2019; Smith and McMurray 2018; Smith et al. 2023). Taken together, these findings suggest that mothers play a leading role in shaping the timing of interactions, providing a scaffolding effect while remaining attuned to their child's vocal behaviour (Kondaurova et al. 2019); children adapt the timing of their vocal behaviours to match that of their mothers, in line with previous work (Smith and McMurray 2018); and the idea that children learn interactional timing through interactions with their caregivers. Importantly, these results from Italian suggest that these phenomena are not specific to English.

### 4.3 Contributions of Home Music Engagement

Our third aim was to investigate, within the CI group one year post‐CI, whether higher levels of home music engagement would be associated with enhanced turn‐taking and timing in the dyad. The results showed no effect of home music engagement on dyads’ responsiveness and simultaneous speech relative frequency. However, we did find an effect on pause duration characteristics: amount of music engagement at home negatively predicted pause mean duration and duration variability. These results indicate that mixed‐hearing dyads who engaged more frequently in musical activities at home produced shorter and less variable pauses in conversation, indicating a smoother and more stable turn‐taking dynamic. These effects support the idea that participation in music activities, though informal, may support listening behaviours as well as synchronization to a shared rhythm between interlocutors (Hidalgo et al. 2017).

The benefits of music engagement may extend beyond timing per se to broader aspects of social interaction. Previous research has shown that joint music making enhances cooperative behaviours and temporal coordination in preschool‐aged children (Beck and Rieser 2020; Kirschner and Tomasello 2010), and that early active musical experience supports not only musical development but also communicative and social skills in infants (Gerry et al. 2012).

Because the positive effect of music engagement on turn‐taking was shown in mixed‐hearing dyads, our results have potentially relevant implications for rehabilitation. In fact, our findings suggest that the interactional timing skills of children with CIs may be improved through involvement in music training programs. Importantly, these benefits may then extend to children's verbal and social development, as previous work has shown that interactional coordination affects children's development of language skills (Masek et al. 2021; Smith et al. 2024) and social relations (Hoffman et al. 2015).

Moreover, our results suggest that turn‐taking is not solely driven by maturational factors, but remains sensitive to variation in the child's auditory and rhythmic environment. In this sense, our findings extend previous work by showing that early conversational timing can be shaped not only by developmental change, but also by experiential factors that either affect the temporal structure of the input (e.g., maternal depression; Smith et al. 2023) or limit children's access to it (e.g., in the case of hearing impairment).

### 4.4 Conclusions

Our study had some limitations, including the relatively small sample size and the lack of analyses on nonverbal cues to examine multimodal communication. Our coding focused exclusively on vocalizations, in continuity with previous work on vocal turn‐taking and pause timing (e.g., Smith and McMurray 2018; Smith et al. 2023), and did not take into account accompanying nonverbal behaviours such as gaze, pointing, or shared visual attention, which are important aspects of communication, especially in children with CIs (e.g., Samuelsson et al. 2025). Future studies may integrate verbal and nonverbal modalities to provide a more comprehensive account of dyadic interaction.

Moreover, in line with previous work on early conversational timing, we defined vocalizations broadly as any non‐vegetative sound, allowing us to capture the full range of vocal behaviours that participate in turn‐taking dynamics, particularly in young children and children with CIs. Future work could include a finer‐grained analysis of the signals produced by children and investigate how different types of vocalizations (speech, protophones, non‐speech vocalizations) affect the timing of conversational coordination.

In addition, in our study we matched the CI and NH groups based on the children's chronological age. However, matching solely on this factor does not fully capture developmental differences between children with CIs and their peers with NH, as they also differ in auditory and language experience. Future research could include a group of children with NH matched to the CI group on auditory experience (i.e., based on the children's hearing age) or language experience (e.g., based on the quantity of input received at home, which could be measured through tools such as LENA; the LENA Research Foundation, Boulder, CO).

Finally, our sample presents high variability in children's age at diagnosis and age at implantation, similarly to previous work in pediatric CI and hearing impairment research (e.g., Dilley et al. 2020; Fagan et al. 2014; Kondaurova et al. 2019; Smith and McMurray 2018). Previous research has shown that both factors (Nicholas and Geers 2007; Yoshinaga‐Itano et al. 1998) have an effect on the spoken language development of children with CIs, and may therefore also influence the timing of their communicative exchanges. However, in our work we did not find evidence of consistent associations between age at diagnosis/implantation and children's interactional characteristics. Future research with larger sample sizes will be necessary to further investigate whether and how age at diagnosis and implantation modulate the timing of conversational coordination in children with CIs.

Nonetheless, our study provides an important contribution to the field by presenting the first longitudinal data on mother–child interactional timing in mixed‐hearing and hearing dyads speaking Italian, fine‐grained temporal information thanks to manual temporal coding, and four key results. First, it showed how turn‐taking and its timing change as children get older and more linguistically proficient, thus highlighting the complex interaction between individual factors, developmental trends, and linguistic proficiency. This phenomenon appears to be universal and not language specific. Second, it showed how some of the characteristics of the interaction are affected by children's hearing impairment. Third, it confirmed previous work showing that mothers and children are coupled in the timing of their vocal behaviours in interaction. Finally, it showed, within the group of children with CIs, that more fluid and stable vocal exchanges between partners were linked to higher music engagement at home, with important implications for research and rehabilitation. In fact, these results suggest that everyday musical experiences may foster the development of communicative timing in early interactions. These insights open new avenues for research into the mechanisms linking music and communication and underscore the potential of music‐based interventions to support language and communicative skills, especially in children at risk for language or communicative difficulties. Further research is warranted to replicate the current findings.

Beyond replicating previously reported developmental and dyadic effects on turn taking in samples speaking different languages, the present findings also highlight that these interactional processes are amenable to environmental influence. In fact, fine‐grained temporal coordination appears to be negatively affected by hearing impairment and positively influenced by music engagement, at least in the group of children with CIs. Future research directly comparing the role of music engagement across hearing status groups will clarify the extent to which musical experiences can positively shape early communicative interactions.

## Funding

This research was supported by the Joint Project Grant 2016 (IIR‐1851), funded by the University of Verona and Cochlear S.r.l. The funding organization had no role in the design and conduct of the study; in the collection, analysis, and interpretation of the data; or in the decision to submit the article for publication; or in the preparation, review, or approval of the article.

## Ethics Statement

The study was approved by the Ethics Committee of the University of Verona (protocol number 2017/46268; P.I.: Marinella Majorano). Mothers signed an informed consent form. The data were treated in accordance with the privacy regulations (Legislative Decree 196/2003).

## Conflicts of Interest

The authors declare no conflicts of interest.

## Supporting information




**Supporting Information**: jlcd70243‐supp‐0001‐SuppMat.docx

## Data Availability

The data that support the findings of this study are available from the corresponding author upon reasonable request.
