# Supplementary material for: Turn‐Taking and Vocal Coordination in Mother–Child Mixed‐Hearing Dyads and the Effect of Home Music Engagement: A Longitudinal Study on Italian Children With Cochlear Implants
Source: Int J Lang Commun Disord. 2026 Apr 17;61:e70243. doi: 10.1111/1460-6984.70243 (PMC13088223; doi:10.1111/1460-6984.70243)
Supplement: Supplementary file 1 — Supporting Information: jlcd70243‐supp‐0001‐SuppMat.docx [file JLCD-61-0-s001.docx]

**Table 1S**

***Full results of the*** ***models on mothers’ and children’s relative frequency or timing of vocal behaviors in conversation***

|  |  | ***Effect or interaction*** | ***F*** | ***df*** | ***p*** |
| --- | --- | --- | --- | --- | --- |
| **Mothers** |  |  |  |  |  |
| **Temporally contingent responses** | Proportion | Group | 5.104 | 1, 32.209 | .031 |
|  |  | Session | 30.309 | 1, 32.099 | <.001 |
|  |  | Group $\times$ Session | 1.269 | 1, 32.099 | .268 |
| **Simultaneous speech (ISS, NSS)** | Proportion | Group | 0.620 | 1, 31.202 | .437 |
|  |  | Session | 12.186 | 1, 87.268 | < .001 |
|  |  | Type | 67.659 | 1, 88.071 | < .001 |
|  |  | Group $\times$ Session | 3.453 | 1, 87.268 | .067 |
|  |  | Group $\times$ Type | 10.596 | 1, 88.071 | .002 |
|  |  | Session $\times$ Type | 8.049 | 1, 86.209 | .006 |
|  |  | Group $\times$ Session $\times$ Type | 2.459 | 1, 86.209 | .121 |
| **Pauses (within, between)** | Mean duration | Group | 0.455 | 1, 30.521 | .505 |
|  |  | Session | 10.346 | 1, 94.026 | .002 |
|  |  | Type | 390.625 | 1, 92.705 | <.001 |
|  |  | Group $\times$ Session | 0.010 | 1, 94.026 | .919 |
|  |  | Group $\times$ Type | 1.009 | 1, 92.705 | .318 |
|  |  | Session $\times$ Type | 0.286 | 1, 92.705 | .594 |
|  |  | Group $\times$ Session $\times$ Type | 0.054 | 1, 92.705 | .817 |
|  | Duration variability (*SD*) | Group | 0.885 | 1, 31.085 | .354 |
|  |  | Session | 6.487 | 1, 94.924 | .013 |
|  |  | Type | 32.124 | 1, 93.467 | <.001 |
|  |  | Group $\times$ Session | 0.159 | 1, 94.924 | .691 |
|  |  | Group $\times$ Type | 0.171 | 1, 93.467 | .680 |
|  |  | Session $\times$ Type | 0.223 | 1, 93.467 | .638 |
|  |  | Group $\times$ Session $\times$ Type | 0.195 | 1, 93.467 | .660 |
| **Children** |  |  |  |  |  |
| **Temporally contingent responses** | Proportion | Group | 1.928 | 1, 31.978 | .175 |
|  |  | Session | 16.464 | 1, 31.642 | <.001 |
|  |  | Group $\times$ Session | 1.073 | 1, 31.642 | .308 |
| **Simultaneous speech (ISS, NSS)** | Proportion | Group | 8.006 | 1, 30.791 | .008 |
|  |  | Session | 21.131 | 1, 92.837 | <.001 |
|  |  | Type | 23.381 | 1, 91.498 | <.001 |
|  |  | Group $\times$ Session | 0.674 | 1, 92.837 | .414 |
|  |  | Group $\times$ Type | 2.727 | 1, 91.498 | .102 |
|  |  | Session $\times$ Type | 0.891 | 1, 91.498 | .348 |
|  |  | Group $\times$ Session $\times$ Type | 0.008 | 1, 91.498 | .929 |
| **Pauses (within, between)** | Mean duration | Group | 2.209 | 1, 32.534 | .147 |
|  |  | Session | 1.122 | 1, 95.742 | .292 |
|  |  | Type | 3.522 | 1, 94.345 | .064 |
|  |  | Group $\times$ Session | 1.884 | 1, 95.742 | .173 |
|  |  | Group $\times$ Type | 0.007 | 1, 94.345 | .932 |
|  |  | Session $\times$ Type | 0.085 | 1, 94.345 | .772 |
|  |  | Group $\times$ Session $\times$ Type | 0.012 | 1, 94.345 | .912 |
|  | Duration variability (*SD*) | Group | 0.624 | 1, 32.523 | .435 |
|  |  | Session | 0.680 | 1, 93.852 | .412 |
|  |  | Type | 11.673 | 1, 92.259 | <.001 |
|  |  | Group $\times$ Session | 1.120 | 1, 93.852 | .293 |
|  |  | Group $\times$ Type | 0.014 | 1, 92.259 | .907 |
|  |  | Session $\times$ Type | 0.057 | 1, 92.340 | .813 |
|  |  | Group $\times$ Session $\times$ Type | 0.164 | 1, 92.340 | .687 |

**Table 2S**

**Full model summaries**

|  |  |  | **Estimate** | ***SE*** | **df** | ***t*** | ***p*** |
| --- | --- | --- | --- | --- | --- | --- | --- |
| **Mothers** |  |  |  |  |  |  |  |
| **Temporally contingent responses** | Proportion | Intercept | 0.192 | 0.032 | 62.315 | 6.036 | < .001 |
|  |  | Group CI | -0.042 | 0.045 | 62.315 | -0.933 | .354 |
|  |  | Session T2 | 0.201 | 0.043 | 31.618 | 4.730 | <.001 |
|  |  | Group NH: Session T2 | -0.068 | 0.061 | 32.099 | -1.127 | .268 |
| **Simultaneous speech (ISS, NSS)** | Proportion | Intercept | 0.057 | 0.011 | 86.071 | 5.258 | < .001 |
|  |  | Group CI | 0.010 | 0.015 | 86.071 | 0.643 | .522 |
|  |  | Session T2 | 0.063 | 0.012 | 85.475 | 5.117 | <.001 |
|  |  | Type NSS | -0.046 | 0.013 | 88.413 | -3.441 | <.001 |
|  |  | Group CI : Session T2 | -0.044 | 0.018 | 85.973 | -2.517 | .014 |
|  |  | Group CI: Type NSS | 0.022 | 0.019 | 87.779 | 1.190 | .237 |
|  |  | Session T2 : Type NSS | -0.057 | 0.018 | 86.595 | -3.101 | .003 |
|  |  | Group CI : Session T2 : Type NSS | 0.040 | 0.026 | 86.209 | 1.568 | .121 |
| **Pauses (within, between)** | Mean duration | Intercept | 1.417 | 0.054 | 111.378 | 26.448 | < .001 |
|  |  | Group CI | -0.062 | 0.076 | 111.378 | -0.824 | .412 |
|  |  | Session T2 | -0.087 | 0.068 | 92.705 | -1.290 | .200 |
|  |  | Type between | -0.680 | 0.068 | 92.705 | -10.079 | < .001 |
|  |  | Group CI : Session T2 | -0.009 | 0.096 | 93.367 | -0.092 | .927 |
|  |  | Group CI : Type between | 0.053 | 0.096 | 92.705 | 0.551 | .583 |
|  |  | Session T2 : Type between | -0.052 | 0.096 | 92.705 | -0.546 | .586 |
|  |  | Group CI : Session T2 : Type between | 0.031 | 0.136 | 92.705 | 0.232 | .817 |
|  | Duration variability (*SD*) | Intercept | 1.017 | 0.052 | 124.338 | 19.698 | < .001 |
|  |  | Group CI | -0.022 | 0.073 | 124.338 | -0.296 | .768 |
|  |  | Session T2 | -0.072 | 0.071 | 93.467 | -1.025 | .308 |
|  |  | Type between | -0.154 | 0.0706 | 93.467 | -2.187 | .031 |
|  |  | Group CI : Session T2 | -0.003 | 0.101 | 94.196 | -0.030 | .976 |
|  |  | Group CI : Type between | -0.061 | 0.100 | 93.467 | -0.610 | .544 |
|  |  | Session T2 : Type between | -0.065 | 0.100 | 93.467 | -0.651 | .517 |
|  |  | Group CI : Session T2 : Type between | 0.063 | 0.142 | 93.467 | 0.442 | .660 |
| **Children** |  |  |  |  |  |  |  |
| **Temporally contingent responses** | Proportion | Intercept | 0.558 | 0.025 | 57.062 | 22.091 | < .001 |
|  |  | Group CI | -0.062 | 0.036 | 57.062 | -1.744 | .087 |
|  |  | Session T2 | 0.063 | 0.029 | 31.211 | 2.159 | .039 |
|  |  | Group CI: Session T2 | 0.043 | 0.042 | 31.642 | 1.036 | .308 |
| **Simultaneous speech (ISS, NSS)** | Proportion | Intercept | 0.168 | 0.022 | 107.163 | 7.671 | < .001 |
|  |  | Group CI | 0.092 | 0.031 | 107.163 | 2.997 | .003 |
|  |  | Session T2 | -0.038 | 0.028 | 91.054 | -1.400 | .165 |
|  |  | Type NSS | -0.030 | 0.028 | 91.939 | -1.074 | .286 |
|  |  | Group CI : Session T2 | -0.025 | 0.039 | 91.687 | -0.650 | .517 |
|  |  | Group CI : Type NSS | -0.048 | 0.039 | 91.940 | -1.229 | .222 |
|  |  | Session T2 : Type NSS | -0.029 | 0.039 | 91.504 | -0.736 | .464 |
|  |  | Group CI : Session T2 : Type NSS | 0.005 | 0.055 | 91.498 | 0.089 | .929 |
| **Pauses (within, between)** | Mean duration | Intercept | 1.154 | 0.076 | 124.063 | 15.081 | < .001 |
|  |  | Group CI | -0.166 | 0.107 | 123.945 | -1.561 | .121 |
|  |  | Session T2 | -0.116 | 0.104 | 95.277 | -1.122 | .265 |
|  |  | Type between | -0.092 | 0.104 | 95.277 | -0.888 | .377 |
|  |  | Group CI : Session T2 | 0.153 | 0.146 | 95.348 | 1.045 | .299 |
|  |  | Group CI : Type between | 0.020 | 0.145 | 94.664 | 0.140 | .889 |
|  |  | Session T2 : Type between | -0.018 | 0.145 | 94.664 | -0.127 | .899 |
|  |  | Group CI : Session T2 : Type between | -0.023 | 0.206 | 94.344 | -0.111 | .912 |
|  | Duration variability (*SD*) | Intercept | 0.837 | 0.077 | 119.677 | 10.869 | < .001 |
|  |  | Group CI | -0.088 | 0.111 | 119.958 | -0.795 | .428 |
|  |  | Session T2 | -0.064 | 0.104 | 93.500 | -0.617 | .539 |
|  |  | Type between | 0.203 | 0.102 | 92.279 | 1.987 | .050 |
|  |  | Group CI : Session T2 | 0.068 | 0.148 | 94.286 | 0.456 | .650 |
|  |  | Group CI : Type between | -0.030 | 0.146 | 92.893 | -0.203 | .839 |
|  |  | Session T2 : Type between | -0.066 | 0.145 | 92.361 | -0.458 | .648 |
|  |  | Group CI : Session T2 : Type between | 0.083 | 0.206 | 92.340 | 0.405 | .687 |
